# Does the 5–2-1 criteria identify patients with advanced Parkinson's disease? Real-world screening accuracy and burden of 5–2-1-positive patients in 7 countries

**DOI:** 10.1186/s12883-022-02560-1

**Published:** 2022-01-24

**Authors:** Irene A. Malaty, Pablo Martinez-Martin, K. Ray Chaudhuri, Per Odin, Matej Skorvanek, Joohi Jimenez-Shahed, Michael J. Soileau, Susanna Lindvall, Josefa Domingos, Sarah Jones, Ali Alobaidi, Yash J. Jalundhwala, Prasanna L. Kandukuri, Koray Onuk, Lars Bergmann, Samira Femia, Michelle Y. Lee, Jack Wright, Angelo Antonini

**Affiliations:** 1 University of Florida, Fixel Institute for Neurological Diseases, Gainesville, FL USA; 2 Center for Networked Biomedical Research in Neurodegenerative Diseases (CIBERNED), Carlos III Institute of Health, Madrid, Spain; 3 Parkinson Foundation Centre of Excellence, King’s College Hospital and King’s College, London, UK; 4 University of Lund, Lund, Sweden; 5 Department of Neurology, P. J. Šafárik University, Košice, Slovakia; 6 Department of Neurology, University Hospital of L. Pasteur, Košice, Slovakia; 7 Department of Neurology, Icahn School of Medicine at Mount Sinai, New York, NY USA; 8 Texas Movement Disorder Specialists, Georgetown, TX USA; 9 European Parkinson’s Disease Association (EPDA), Sevenoaks, UK; 10 Grupo de patologia médica, nutrição e exercício clínico (PaMNEC) do CiiEM, Almada, Portugal; 11 Parkinson & Movement Disorder Alliance, Tucson, USA; 12 AbbVie Inc., North Chicago, IL USA; 13 University of Illinois at Chicago, Chicago, IL USA; 14 Adelphi Real World, Bollington, UK; 15 Parkinson and Movement Disorders Unit, Department of Neuroscience, University of Padua, Padova, Italy

**Keywords:** Advanced Parkinson’s disease, 5–2-1 criteria, Screening performance, clinical burden

## Abstract

**Background:**

The burden of Parkinson’s disease (PD) worsens with disease progression. However, the lack of objective and uniform disease classification challenges our understanding of the incremental burden in patients with advanced Parkinson’s disease (APD) and suboptimal medication control. The 5–2-1 criteria was proposed by clinical consensus to identify patients with advancing PD. Our objective was to evaluate the screening accuracy and incremental clinical burden, healthcare resource utilization (HCRU), and humanistic burden in PD patients meeting the 5–2-1 screening criteria.

**Methods:**

Data were drawn from the Adelphi Parkinson’s Disease Specific Program (DSP™), a multi-country point-in-time survey (2017–2020). People with PD who were naive to device-aided therapy and on oral PD therapy were included. Patients meeting the 5–2-1 screening criteria had one or more of the three clinical indicators of APD: (i) ≥5 doses of oral levodopa/day, OR (ii) “off” symptoms for ≥2 h of waking day, OR (iii) ≥1 h of troublesome dyskinesia. Clinician assessment of PD stage was used as the reference in this study. Clinical screening accuracy of the 5–2-1 criteria was assessed using area under the curve and multivariable logistic regression models. Incremental clinical, HCRU, and humanistic burden were assessed by known-group comparisons between 5 and 2-1-positive and negative patients.

**Results:**

From the analytic sample (*n* = 4714), 33% of patients met the 5–2-1 screening criteria. Among physician-classified APD patients, 78.6% were 5–2-1 positive. Concordance between clinician judgment and 5–2-1 screening criteria was > 75%. 5–2-1-positive patients were nearly 7-times more likely to be classified as APD by physician judgment. Compared with the 5–2-1-negative group, 5–2-1-positive patients had significantly higher clinical, HCRU, and humanistic burden across all measures. In particular, 5–2-1-positive patients had 3.8-times more falls, 3.6-times higher annual hospitalization rate, and 3.4-times greater dissatisfaction with PD treatment. 5–2-1-positive patients also had significantly lower quality of life and worse caregiver burden.

**Conclusions:**

5–2-1 criteria demonstrated potential as a screening tool for identifying people with APD with considerable clinical, humanistic, and HCRU burden. The 5–2-1 screening criteria is an objective and reliable tool that may aid the timely identification and treatment optimization of patients inadequately controlled on oral PD medications.

**Supplementary Information:**

The online version contains supplementary material available at 10.1186/s12883-022-02560-1.

## Background

Parkinson’s disease (PD) is one of the fastest growing neurological disorders worldwide, with a reported 118% increase in prevalence between 1990 and 2015 [1, 2]. In 2015, approximately 6.2 million individuals globally were estimated to have PD [1, 2].

As PD progresses, patients tend to experience greater disability and an impaired ability to perform activities of daily living (ADLs) [3, 4], as well as fluctuating response to standard oral PD therapies [4] and delayed gastric emptying [5] leading to poorer symptom control. In order to achieve optimal PD symptom control, patients may require advanced treatment options such as deep brain stimulation, subcutaneous apomorphine infusion, and levodopa–carbidopa intestinal gel (LCIG) [6].

Unfortunately, progress in APD management has been impeded by a lack of objective and standardized diagnostic criteria to identify patients with advanced disease in clinical practice. This is further compounded by the complexity and heterogeneity of PD symptoms, making timely and accurate patient identification even more challenging [4]. A uniform set of criteria for rapidly identifying APD is therefore urgently required to screen for patients with suboptimal symptom control [4, 6–8].

A number of different screening and management approaches have previously been used in an attempt to identify APD, including the use of biomarkers [9, 10] and uni- and multi-dimensional scales such as the Hoehn and Yahr (H&Y) scale [11], the Parkinson Disease Composite Scale (PDCS) [12], and the *Cuestionario De Enfermedad de Parkinson Avanzada* (Questionnaire for Advanced Parkinson’s Disease; CDEPA) [13]. In addition, the Unified Parkinson’s Disease Rating Scale (UPDRS) is widely used to follow the course of PD [14], but cannot be used to truly diagnose APD as outcomes may vary according to the time of the clinical evaluation (i.e., whether the patient was in the ON or OFF state).

An alternative approach to screening for APD has recently emerged from a consensus group tasked with assessing clinical indicators of APD [4, 15] in a modified Delphi study. The consensus group agreed on 15 clinically relevant indicators (six motor symptoms [MS], five non-motor symptoms [NMS], and four functional impairments) for suspected APD [4]. Of the 15 identifiers described, a combination of three key criteria was later proposed as a simple-to-use screening tool to identify patients with suspected APD [4, 16, 17], namely: i) taking ≥5 doses of oral levodopa per day, or ii) having ‘off’ symptoms for ≥2 h of waking day, or iii) having ≥1 h of troublesome dyskinesia per waking day. Under this proposal, patients meeting at least one of these three screening criteria (‘5–2-1 positive’ patients) would be considered as potentially having advanced disease.

Previous studies have suggested the potential utility of the 5–2-1 criteria as a screening tool for APD [17, 18]. In one study, individuals meeting that 5–2-1 criteria were found to have significantly poorer health outcomes, including worse quality of life (QoL), greater NMS burden, and greater caregiver burden [16]. In a retrospective analysis of the DUOGLOBE study, 98% of patients who had been selected for LCIG by investigators were found to fulfill at least one of the 5–2-1 criteria, suggesting that the 5–2-1 criteria apply to a population of patients identified by clinicians as having APD [19].

However, the few studies evaluating the 5–2-1 screening criteria have tended to be small, single-centered investigations offering limited evidence on real-world screening accuracy of the 5–2-1 criteria and little information on the incremental burden experienced by 5–2-1-positive patients [18]. To improve our understanding of the utility of the 5–2-1 screening criteria in every day clinical practice, the current real-world analysis was undertaken. The objectives of this study were two-fold: to evaluate the screening accuracy of the 5–2-1 criteria in identifying people with APD, and to quantify the incremental clinical and humanistic burden as well as the healthcare resource utilization (HCRU) of 5–2-1-positive patients.

## Methods

Data for this retrospective study were drawn from the Adelphi Parkinson’s Disease Specific Program (DSP™), a point-in-time survey of physicians and their consulting PD patients presenting in a real-world clinical setting. The DSP was conducted in G7 countries (France, Germany, Italy, Japan, Spain, the UK, and the USA) between 2017 and 2020. The DSP methodology has been previously published and validated [20]. Participating physicians completed a record form for the next 12 consecutively consulting patients meeting the eligibility criteria; the physician-reported questionnaire form contained questions on patients’ demographics, clinical assessments, clinical outcomes, medication use and history, healthcare resource utilization, and concomitant conditions. Each patient for whom the physician completed a form was then invited to complete a patient-reported questionnaire, which recorded demographics, current condition, level of satisfaction with their treatment, and QoL. If the patient had a caregiver, they were also invited to complete a form, reporting on demographics, and burden of care.

Respondents provided informed consent for use of their data; all data were aggregated and de-identified before receipt.

### Participants

Physicians were eligible to participate in the survey if they were a neurologist, and they were personally responsible for treatment decisions and management of patients with PD. Patients were eligible for inclusion in the survey if they were diagnosed with PD on or before the date of their last consultation (i.e., at the time of data collection), and aged ≥18 years. Patients were eligible for inclusion in this analysis if they were taking oral therapy for PD, and naive to treatment with device-aided therapy (DAT).

### Measures

Patients meeting the 5–2-1 screening criteria were identified as those experiencing one or more of the following: (i) taking ≥5 doses of oral levodopa per day, OR (ii) having ‘off’ symptoms for ≥2 h of waking day, OR (iii) having ≥1 h of troublesome dyskinesia per waking day. The physician’s opinion of disease severity (defined as APD vs non-advanced PD), based on overall patient history including demographics, clinical characteristic, concomitant medications, and other patient-reported outcomes, was used as the reference for this study. As all participating physicians had to see a minimum of three PD patients per week, all were considered highly experienced and can reasonably be expected to reliably distinguish degree of PD severity.

We also captured clinical, HCRU, and humanistic PD burden to quantify the burden of patients meeting the 5–2-1 screening criteria. Clinical burden was evaluated based on the following measures: UPDRS (clinical rating scale for PD encompassing behavior and mood, ADLs, MS, and complications; higher score indicates greater burden) [21], Mini-Mental State Examination (MMSE; 11-question instrument testing five areas of cognitive function; lower score indicates worse cognitive function) [22], Charlson comorbidity index (based on a number of conditions that are each assigned an integer weight from one to six; higher score indicates greater comorbidity burden) [23], ADL (assessed as percentage of patients able to carry out specific ADLs independently), and falls (mean number of PD-related falls in the last 12 months, based on patient report). HCRU was assessed by considering the following measures: hospitalization rate (number and percentage of patients hospitalized in the last 12 months for PD, including surgical procedures), physician consultations (number of consultations with a physician in the last 12 months, as captured on patient records), and hours of caregiver help (total number of hours of caregiver help required per week, including professional and non-professional support). Humanistic burden was based on following measures: Parkinson’s Disease Questionnaire (PDQ-39; questionnaire assessing PD-related difficulties in daily living across eight dimensions; higher score indicates greater burden) [24], EuroQol 5 Dimension (EQ-5D; a generic instrument to assess health-related QoL; scores range from 0 to 1, with a lower score indicating poorer QoL) [25], PD treatment satisfaction (number and percentage of patients reporting satisfaction with their drug treatment [measured on a patient-rated 7-point scale] with their drug treatment), and Zarit Burden Interview (ZBI; caregiver self-report 29-item questionnaire; higher score indicates greater burden) [26].

### Statistical analysis

Multivariable logistic regression models were computed to evaluate the screening performance of the 5–2-1 criteria as a predictor and clinician assessment of APD as an outcome. The screening performance of the 5–2-1 criteria was evaluated based on the following: odds ratio (OR) and 95% confidence interval (CI), correct classification percentage, and area under the curve (AUC). Correct classification is the percentage of patients correctly classified per 5–2-1 criteria (sum of true positive and true negatives divided by total number of patients), and is described using the term ‘concordance’. AUC is a screening accuracy measure that balances sensitivity (i.e., presence of the 5–2-1 screening criteria in patients with APD according to physician’s judgment) and specificity (i.e., absence of the 5–2-1 screening criteria in patients with non-advanced PD). AUC is interpreted as follows: AUC = 0.5 suggests a non-informative result (no better than chance); AUC = 0.5 to ≤0.7 is considered less accurate; AUC = 0.7 to ≤0.9 is considered moderately accurate, AUC 0.9 to < 1 is considered highly accurate, and AUC = 1 is considered the perfect test [27–29]. All logistic regression models were adjusted for potential confounders including country, patient age (< 65 years or ≥ 65 years), gender, time since PD diagnosis, and Charlson comorbidity index.

Incremental clinical burden, healthcare resource utilization, and humanistic burden was evaluated based on known-group comparisons between 5 and 2-1 positive and 5–2-1-negative groups using Wilcoxon-Mann-Whitney, t-test, chi-square, and Fisher’s exact tests, as appropriate.

## Results

### Sample characteristics

In total, 4714 patients were included from all G7 countries (France: 10.5%, Germany: 12.6%, Italy: 11.3%, Japan: 11.9%, Spain: 11.6%, UK: 12.4%, and USA: 29.6%). The mean (standard deviation, SD) time since diagnosis was 4.3 (4.4) years, and 33.5% of patients were H&Y stage 3–5 in the “on” state. Of the 4714 patients included, 14.9% (*n* = 702) were classified by the physician as having APD.

According to the criteria described above, 32.8% of patients (*n* = 1546) were 5–2-1 positive, including 552 patients who had been classified by the physician as having APD (78.6% of all APD patients). Compared with 5–2-1-negative patients, 5–2-1-positive patients were older, had a longer time since diagnosis, had a higher H&Y stage, and were more often classified as having APD by their physician (Table 1). 5–2-1 positive patients had a higher Charlson Comorbidity Index, the observed difference being informed by a greater prevalence of cardiovascular conditions, diabetes, and dementia (Supplementary Table 1).

**Table 1:** Clinical characteristics of 5–2-1-positive and 5–2-1-negative patients

	All patients (***n*** = 4714)^**a**^	5–2-1 positive (***n*** = 1546)^**a**^	5–2-1 negative (***n*** = 3168)^**a**^	***P***-value
Patient age				
Mean (SD)	69.3 (10.6)	71.7 (10.4)	68.2 (10.5)	< 0.0001
≥65 years, n (%)	3309 (70.2)	1231 (79.6)	2078 (65.6)	< 0.0001
Gender, n (%)				
Male	2866 (60.8)	964 (62.4)	1902 (60.0)	0.1274
Charlson Comorbidity Index, mean (SD)	0.4 (1.0)	0.6 (1.2)	0.3 (0.9)	< 0.0001
Charlson Comorbidity Index, n (%)				
None (score 0)	3765 (79.9)	1108 (71.7)	2657 (83.9)	< 0.0001
Mild (score 1–2)	787 (16.7)	357 (23.1)	430 (13.6)	
Moderate (score 3–4)	120 (2.5)	62 (4.0)	58 (1.8)	
Severe (score 5+)	42 (0.9)	19 (1.2)	23 (0.7)	
Time since PD diagnosis (years), mean (SD)	4.3 (4.4) (*n* = 3712)	7.2 (5.1) (*n* = 1162)	3.0 (3.2) (*n* = 2550)	< 0.0001
Physician judgment of PD stage, n (%)				
Non-advanced	4012 (85.1)	994 (64.3)	3018 (95.3)	< 0.0001
Advanced	702 (14.9)	552 (35.7)	150 (4.7)	
Hoehn and Yahr stage, n (%)				
1	1593 (33.8)	1579 (39.4)	14 (2.0)	< 0.0001
2	1545 (32.8)	1492 (37.2)	53 (7.5)	
3	932 (19.8)	778 (19.4)	154 (21.9)	
4	457 (9.7)	147 (3.7)	310 (44.2)	
5	187 (4.0)	16 (0.4)	171 (24.4)	

*PD* Parkinson’s disease, *SD* Standard deviation

^a^Patient number unless otherwise stated

### Accuracy

An evaluation of the screening accuracy of the 5–2-1 criteria and clinical indicators of APD with respect to the physician diagnosis is summarized in Table 2. The 5–2-1 screening criteria demonstrated moderate screening accuracy for APD, with AUC values ≥0.80. A comparable analysis carried out using H&Y stages (H&Y stage 1–2 vs H&Y stage 3–5) in place of physician judgement, and the outcome was similar (moderate screening accuracy for APD) (Supplementary Table 2).

**Table 2:** Screening accuracy of the 5–2-1 screening criteria in identifying patients with advanced Parkinson’s disease

	Advanced PD		Adjusted Model ^**a**^		
Indicator	No	Yes	OR (95% CI)	Correct Classification (%) ^**b**^	AUC ^**c**^
5–2-1 screening criteria					
Negative	3018	150	1	–	–
Positive	994	552	6.84 (5.50, 8.51)	88.1	0.89
Individual clinical indicators					
≥2 h off-time/day					
Negative	3299	208	1	–	–
Positive	713	494	7.07 (5.76, 8.68)	88.4	0.89
≥1 h troublesome dyskinesia/day					
Negative	3944	591	1	–	–
Positive	68	111	5.56 (3.85, 8.02)	87.1	0.85
≥5 doses of oral levodopa/day					
Negative	3501	413	1	–	–
Positive	511	289	3.03 (2.45, 3.73)	87.1	0.86

^a^Regressions adjusted for age, sex, time since diagnosis, Charlson Comorbidity Index, and country. ^b^Correct classification is the percentage of patients correctly classified per 5–2-1 criteria (sum of true positive and true negatives divided by total number of patients). ^C^ AUC is a screening accuracy measure that balances sensitivity and specificity. AUC is interpreted as follows: AUC = 0.5, non-informative; AUC = 0.5 to ≤0.7, less accurate; AUC = 0.7 to ≤0.9, moderately accurate, AUC 0.9 to < 1, highly accurate; AUC = 1, perfect test [27–29]

*AUC* Area under the curve, *CI* Confidence interval, *OR* Odds ratio

From the multivariable logistic regression, 5–2-1-positive patients were nearly 7 times more likely to be classified as having APD than 5–2-1-negative patients (adjusted OR 6.84; 95% CI, 5.50–8.51). Patients taking ≥5 oral levodopa doses/day were over 3 times more likely to be classified as having APD, patients having ≥2 h of ‘off’-time/waking day were over 7 times as likely, and patients having ≥1 h of troublesome dyskinesia/waking day were 5 times more likely to be classified as having APD.

There was a minority of patients for whom clinician judgment and 5–2-1 screening criteria did not align, with 150 (3.2%) 5–2-1-negative patients classified as advanced PD and 994 (21.1%) 5–2-1-positive patients classified as non-advanced PD by the clinician. However, overall concordance was greater than 75%. Further analysis revealed that the small group of false negatives (clinician-defined APD patients who were 5–2-1 negative) tended to be older, diagnosed at a later age, and had higher trend for NMS compared with 5–2-1-positive patients classified as having non-advanced disease (Supplementary Table 3).

### Clinical burden

**Fig. 1:**
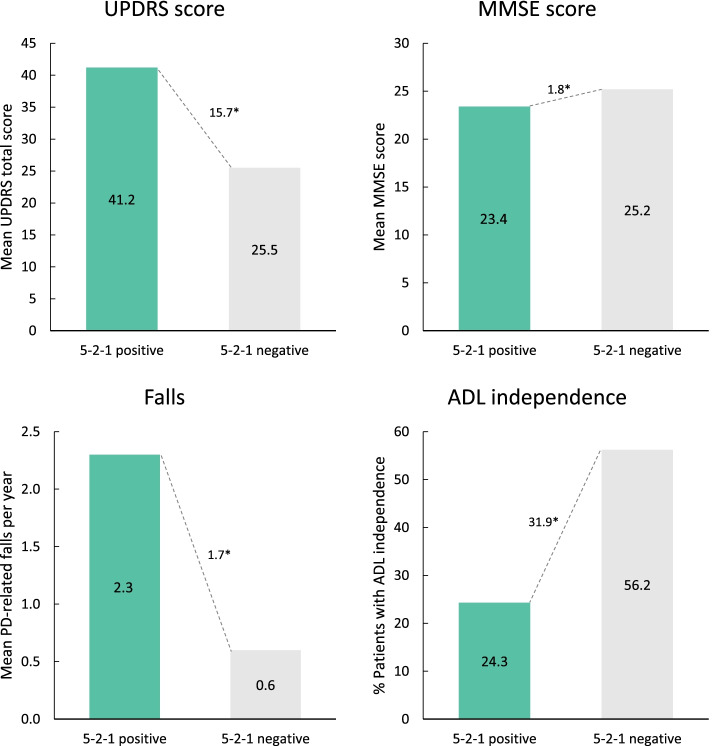
Measures of clinical burden in 5–2-1-positive patients compared with 5–2-1-negative patients. *p < 0.0001. 5–2-1, taking ≥5 doses of oral levodopa/day, OR having ≥2 h ‘off’-time/waking day, OR having ≥1 h troublesome dyskinesia/waking day. ADL, activities of daily living; MMSE, Mini-Mental State Examination; PD, Parkinson’s disease; UPDRS, Unified Parkinson’s Disease Rating Scale

**Fig. 2:**
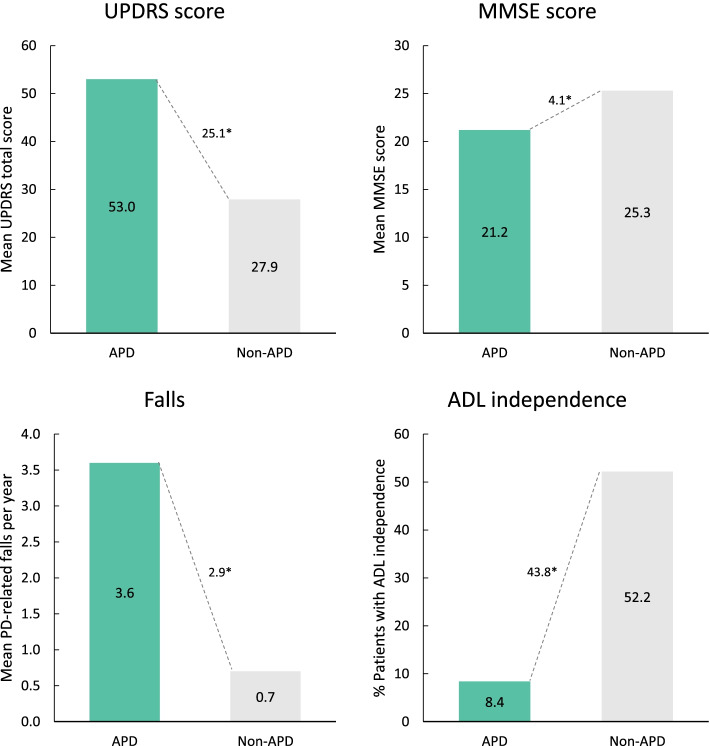
Measures of clinical burden in APD patients compared with non-APD patients (physician judgement). *p < 0.0001. ADL, activities of daily living; APD, advanced Parkinson’s disease; MMSE, Mini-Mental State Examination; PD, Parkinson’s disease; UPDRS, Unified Parkinson’s Disease Rating Scale

Clinical burden across all measures assessed was significantly greater in the 5–2-1-postive group compared with 5–2-1-negative patients (Fig. 1). In particular, 5–2-1-positive patients had higher UPDRS total score (∆ + 15.7 [41.2 vs 25.5; *p* < 0.0001]) and lower MMSE score (∆ -1.8 [23.4 vs 25.2; *p* < 0.0001]), indicating a greater clinical burden compared with 5–2-1-negative patients. Patients in the 5–2-1-positive group had experienced more PD-related falls in the last 12 months (∆ + 1.7 falls [2.3 vs 0.6 falls; *p* < 0.0001]) compared with 5–2-1-negative patients. Significantly fewer 5–2-1-positive patients showed independence in carrying out ADLs (24.3% vs. 56.2%; *p* < 0.0001). Similar findings were seen when data were analyzed according to physician judgement (Fig. 2) or H&Y stage (Supplementary Fig. 1).

### Healthcare resource utilization burden

**Fig. 3:**
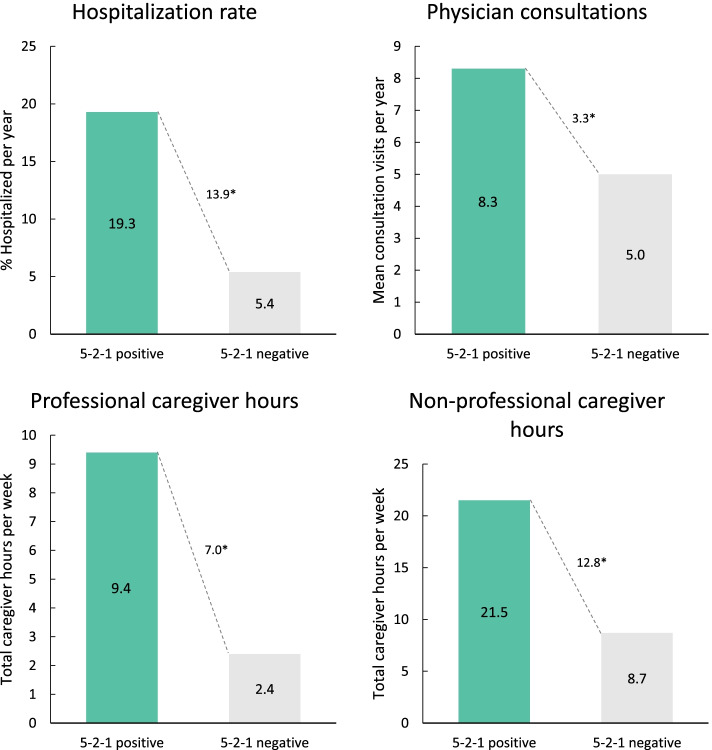
Measures of healthcare resource utilization in 5–2-1-positive patients compared with 5–2-1-negative patients. *p < 0.0001. 5–2-1, taking ≥5 doses of oral levodopa/day, OR having ≥2 h ‘off’-time/waking day, OR having ≥1 h troublesome dyskinesia/waking day

**Fig. 4:**
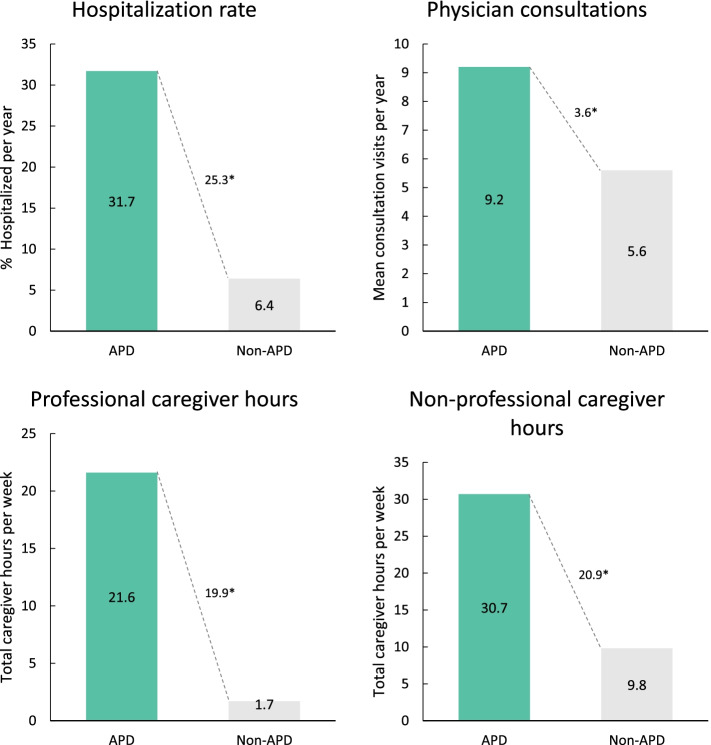
Measures of healthcare resource utilization in APD patients compared with non-APD patients (physician judgement). *p < 0.0001. APD, advanced Parkinson’s disease

Healthcare resource utilization was significantly greater among 5–2-1-positive patients than 5–2-1-negative patients across all measures assessed (Fig. 3). In particular, considerably more patients in the 5–2-1-positive group (19.3% vs 5.4%; *p* < 0.0001) were hospitalized in the last 12 months. 5–2-1-positive patients had a significantly higher number of annual physician consultations (∆ + 3.3 consults/year [8.3 vs 5.0 consults/year; *p* < 0.0001]) and required a significantly greater amount of professional and non-professional caregiver support (∆ + 19.7 h/week overall [30.8 vs 11.1 h/week; *p* < 0.0001]). Similar findings were seen when data were analyzed according to physician judgement (Fig. 4) or H&Y stage (Supplementary Fig. 2).

### Humanistic burden

**Fig. 5:**
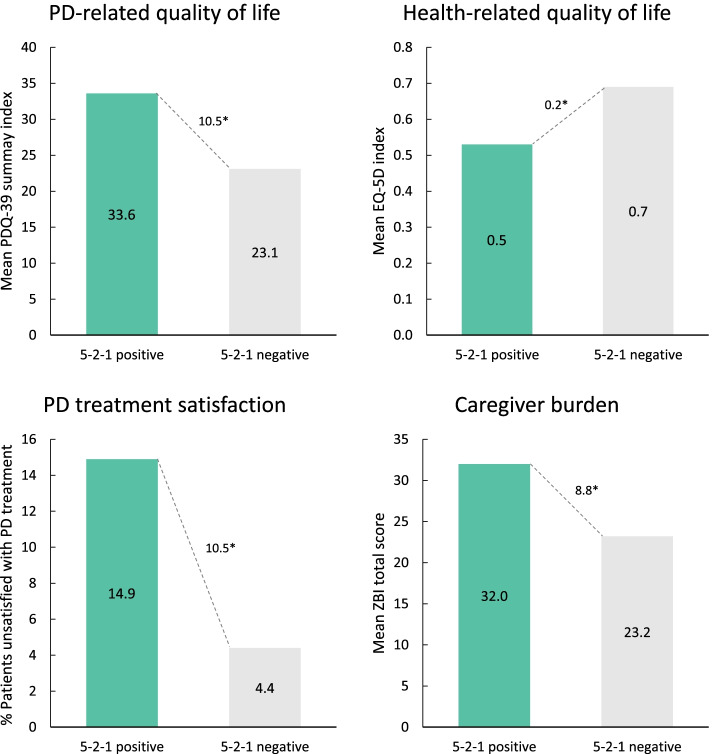
Measures of humanistic burden in 5–2-1-positive patients compared with 5–2-1-negative patients. *p < 0.0001. 5–2-1, taking ≥5 doses of oral levodopa/day, OR having ≥2 h ‘off’-time/waking day, OR having ≥1 h troublesome dyskinesia/waking day. EQ-5D, EuroQol 5-Dimension (1.0 indicates best QoL); PD, Parkinson’s disease; PDQ-39, Parkinson’s Disease Questionnaire-39 (higher score indicates worse QoL); ZBI, Zarit Burden Interview

**Fig. 6:**
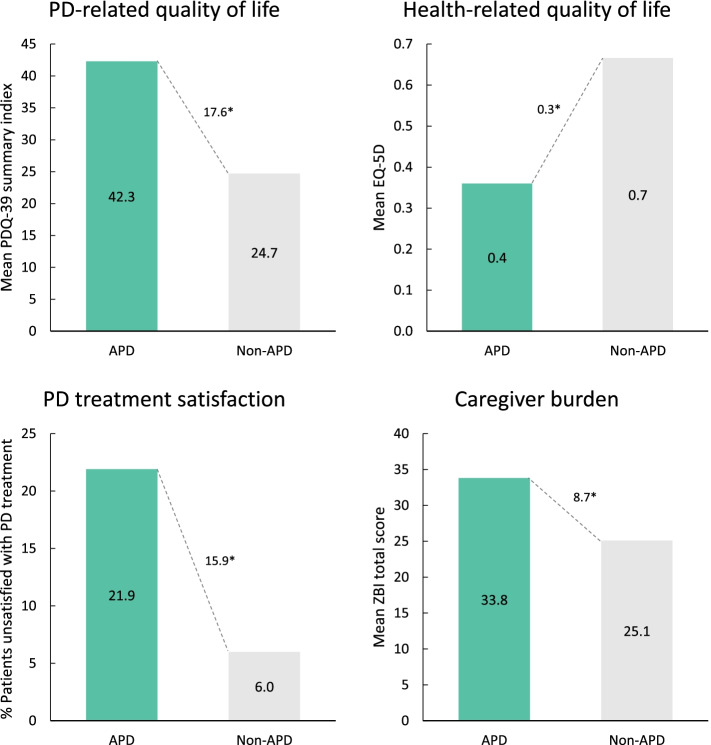
Measures of humanistic burden in APD patients compared with non-APD patients (physician judgement). *p < 0.0001. APD, Advanced Parkinson’s disease; EQ-5D, EuroQol 5-Dimension (1.0 indicates best QoL); PD, Parkinson’s disease; PDQ-39, Parkinson’s Disease Questionnaire-39 (higher score indicates worse QoL); ZBI, Zarit Burden Interview

Humanistic burden across all measures assessed was significantly worse among 5–2-1-positive patients (Fig. 5). Compared with the 5–2-1-negative group, 5–2-1-positive patients had a significantly higher PDQ-39 index score (∆ + 10.5 [33.6 vs 23.1; *p* < 0001]) and lower EQ-5D utility score (∆ -0.16 [0.53 vs 0.69; *p* < 0.0001]), indicating a greater burden on PD and health-related QoL among 5–2-1-positive patients. A significantly greater proportion of patients in the 5–2-1-positive group were reported to be unsatisfied with their drug treatment compared with 5–2-1-negative patients (14.9% vs 4.4%; *p* < 0.0001). Caregivers of 5–2-1-positive patients also demonstrated worse caregiver burden based on ZBI total score (∆ + 8.8 [32.0 vs 23.3; *p* < 0.0001]). Similar findings were seen when data were analyzed according to physician judgement (Fig. 6) or H&Y stage (Supplementary Fig. 3).

## Discussion

To date, this is the largest study to have evaluated the performance of the 5–2-1 criteria in screening and evaluating patients with APD. The 5–2-1 screening criteria demonstrated potential as a screening tool for identifying people with APD. Patients classified as 5–2-1-positive while receiving oral PD treatment had incrementally higher clinical, humanistic, and HCRU burden compared with the 5–2-1-negative group. Our data may suggest the potential of the 5–2-1 criteria as a screening tool to help recognize people with APD who are sub-optimally controlled on oral PD treatment in the real-world [16, 19]. Our results also reinforce the need to identify current unmet needs among these patients due to their increased disease burden.

Although effective disease management is key at all stages of PD, the need for greater individual customization becomes increasingly important as the disease advances [30]. A number of previous attempts have been made to devise simple screening tools that can rapidly identify people with APD who would benefit from further monitoring and optimization of their care plan. In addition, management tools have been developed to evaluate PD symptoms, guide treatment protocols, and ensure that patients with the greatest burden receive optimal treatment. In terms of rapid screening for APD, the duration of disease has traditionally been the first characteristic to be considered. However, definitions of ‘long’ disease duration vary among studies [31–34]. Symptoms do not progress linearly or uniformly over time, and deteriorate at a different pace in different patients. For these reasons, a long duration of disease does not necessarily correspond to more advanced disease [35]. Tools designed to guide the management of PD have tended to include instruments based on specific clinical criteria [10, 13, 15, 16], the use of ratings scales such as the H&Y or UPDRS [14, 36], and the use of biomarkers [37]. However, the universal application of these tools is limited by the substantial time and resources required to apply them.

New approaches have been developed over recent years as we recognized the clear need for improved screening and management tools. One example is an algorithm that was devised to screen for APD based on any 30-day average levodopa equivalent dose (LED) > 1000 mg/day [3]. When this algorithm was applied, approximately 20% of the sample was classified as having advanced disease. However, this algorithm is specific to dopaminergic therapies and does not capture other motor or non-motor aspects of APD, while some patients with a low body mass index may never reach a LED dose > 1000 mg/day despite having advanced disease. In a similar approach, Weir and colleagues used LED > 1100 mg/day as one of several proxy markers of APD in a study of PD-related economic costs in the UK, alongside dyskinesias, falls, dementia, psychosis, hospital admission primarily due to PD, and nursing home placement. This study showed an increased economic burden of PD among patients meeting these markers of advanced disease [38].

The results of our study suggest that the 5–2-1 screening criteria would be a useful method for screening patients with advanced disease who may require more tailored assessment and treatment. Having easily identified people with PD who have a greater burden (5–2-1-positive patients), more comprehensive management instruments (i.e., Making Informed Decisions to Aid Timely Management of Parkinson’s Disease [MANAGE-PD] tool) [39] can be subsequently adopted to ensure further optimization of treatment. Despite recent advances in the development of screening and management tools, an urgent need remains for a screening tool that is simple to use and remember, is based on readily available information, and that clearly identifies patients with a greater disease burden who may benefit from treatment optimization, including a possible second-line therapy.

The MANAGE-PD is a validated tool designed to support clinician decision-making and facilitate standardized and comprehensive assessment of PD-related symptoms [39]. The 5–2-1 criteria aligns with 3 out of 5 key items assessed by MANAGE-PD section 1 to identify patients with inadequate symptoms control. MANAGE-PD section 1 also includes additional questions relating to severity unpredictable fluctuations of motor symptoms and limitations in activities of daily living, making it slightly longer to use, but more comprehensive in its assessment. In a similar way anticipated for the 5–2-1 criteria, MANAGE-PD section 1 groups patients into controlled or inadequately controlled on current therapy.

A recently-reported study compared the 5–2-1 criteria to a newly developed screening tool designed to predict eligibility for referral for DAT evaluation [18]. In this study, a panel of experts assessed 259 consecutive DAT-naive PD patients, of whom 17 were considered eligible for DAT. The study reported several strong predictors of eligibility for referral for DAT, including the presence of response fluctuations and troublesome dyskinesias. Based on their findings, the authors constructed a three-factor screening tool based on LED, response fluctuations, and troublesome dyskinesias, to screen for patients eligible for DAT referral. This study reported a sensitivity and a specificity of 88 and 98% compared with 94 and 73%, respectively, for the 5–2-1 criteria [18]. The authors note that their tool corroborates the concepts of the 5–2-1 criteria, and the study appears to confirm the validity of the overall approach. In this regard, the 5–2-1 concept has identified a new way to think about DAT-eligible patients and has motivated clinicians to find ways to enhance identification.

One limitation of our study was the acceptance of the physician’s opinion of disease severity as the reference against which the 5–2-1 screening criteria were assessed. However, as physician judgement was informed based on the overall condition of the patient, this was considered the best available proxy for management in clinical practice. Furthermore, the categorization of a patient in practice may depend on the experience of the physician, as well as heterogeneous features of PD, including NMS which are not encompassed in 5–2-1. However, although the 5–2-1 screening criteria are driven by MS, there is also a high correlation with other factors, such that patients with advanced or severe MS are also likely to experience NMS [40, 41]. It is also worth noting that the rate of potential APD patients identified through the 5–2-1 screening criteria in this study (33%) was higher than that identified by physicians (15%), thus reinforcing the potential value of the 5–2-1 criteria as a practical screening tool mandating further patient investigation, rather than a diagnostic tool in its own right. Similar findings were reported by Martinez-Martin et al., where the number of APD patients identified with the CDEPA questionnaire was higher compared to clinician judgment [13]. The CDEPA questionnaire is a screening tool that includes measures of both MS and NMS.

The reliance on patient reports to capture data on falls and number of physician consultations is also a potential limitation of this study. However, although self-report has clear limitations, potential inaccuracies may impact both groups (5–2-1 positive and 5–2-1 negative), while both measures are used as markers of unmet need, rather than for precise quantification. A further limitation of this study is that the only aspects of HCRU considered were hospitalization rate, physician consultations, and amount of caregiver help; meaning that the important contributions of other healthcare professionals such as nurses, physiotherapists and occupational/speech therapists were not captured. In addition, we were not able to evaluate the full economic burden of PD, including indirect costs of treatment and productivity losses. All aspects of the economic burden require further consideration in future studies. Our study also has the limitations inherent in point-in-time studies, including the risk of bias from the absence of randomization and/or due to physician, patient, and caregiver recall limitations. However, this large, international dataset is derived from the Parkinson’s DSP, which has been validated for capturing large, statistically robust samples of global real-world evidence, while the data collected reflect current clinical practice. Future studies should evaluate the potential impact of timely escalation in therapy, which may include DAT, to alleviate the disease burden in patients meeting the 5–2-1 screening criteria.

Some consideration should also be given to ‘discordant’ patients, in whom clinician judgment and 5–2-1 screening criteria did not align. It is interesting that the majority of these patients (21.1%) were 5–2-1 positive and non-advanced per their physicians, which is consistent with the potential utility of the 5–2-1 criteria as an early screening tool for advancing PD. In addition, the 5–2-1-negative and clinician-defined advanced group were more likely to have dementia and other cognitive/behavioral symptoms of PD (including confusion, short-term memory loss, difficulties planning, and hallucinations), suggesting that NMS are a significant burden in this patient group. As noted above, NMS are not captured by the 5–2-1 screening criteria, which may explain why a small number of patients whose advanced disease was driven by NMS may have been 5–2-1 negative.

## Conclusions

This study demonstrated the robust screening performance of the 5–2-1 screening criteria in recognizing patients with APD who are sub-optimally controlled on oral PD treatment. This evidence also highlighted the increased clinical, humanistic, and HCRU burden experienced by 5–2-1 positive patients. In particular, the 5–2-1 screening criteria represent a tool that is simple to use and remember, uses readily available information, and demonstrates clinical value in identifying patients with suboptimal disease control, worse QoL, and higher caregiver burden. The 5–2-1 screening criteria provide an objective and reliable tool that may aid in the timely identification and treatment optimization of patients who are inadequately controlled on oral PD medications.

## Supplementary Information

**Additional file 1.**

## Data Availability

The datasets generated and/or analyzed during the current study are not publicly available as they are the intellectual property of Adelphi Real World but are available from the corresponding author on reasonable request.

## Abbreviations

ADL: Activities of daily living
APD: Advanced Parkinson’s disease
AUC: Area under the curve
CI: Confidence interval
DAT: Device-aided therapy
DSP: Disease Specific Program™
EQ-5D: EuroQol 5 Dimension
H&Y: Hoehn and Yahr scale
LCIG: Levodopa–carbidopa intestinal gel
LED: Levodopa equivalent dose
MANAGE-PD: Making Informed Decisions to Aid Timely Management of Parkinson’s Disease
MMSE: Mini-Mental State Examination
MS: Motor symptoms
NMS: Non-motor symptoms
OR: Odds ratio
PD: Parkinson’s disease
PDCS: Parkinson Disease Composite Scale
PDQ-39: Parkinson’s Disease Questionnaire-39
QoL: Quality of life
SD: Standard deviation
UPDRS: Unified Parkinson’s Disease Rating Scale
ZBI: Zarit Burden Interview
