# Supplementary material for: Does the 5–2-1 criteria identify patients with advanced Parkinson's disease? Real-world screening accuracy and burden of 5–2-1-positive patients in 7 countries
Source: BMC Neurol. 2022 Jan 24;22:35. doi: 10.1186/s12883-022-02560-1 (PMC8785442; doi:10.1186/s12883-022-02560-1)
Supplement: Supplementary file 1 — Additional file 1. [file 12883_2022_2560_MOESM1_ESM.docx]

**Supplementary Table 1: Patient comorbidities from the Charlson Comorbidity Index**

|  | **Overall**  **(N=4714)** | **5-2-1-positive**  **(N=1546)** | **5-2-1-negative**  **(N=3168)** | **p-value** |
| --- | --- | --- | --- | --- |
| Comorbidities, n (%) |  |  |  |  |
| Myocardial infarction | 283 (6.0) | 123 (8.0) | 160 (5.1) | 0.0001 |
| Congestive heart failure | 227 (4.8) | 113 (7.3) | 114 (3.6) | <0.0001 |
| Peripheral vascular disease | 170 (3.6) | 80 (5.2) | 90 (2.8) | <0.0001 |
| Cerebrovascular disease | 290 (6.2) | 138 (8.9) | 152 (4.8) | <0.0001 |
| Dementia | 365 (7.7) | 244 (15.8) | 121 (3.8) | <0.0001 |
| Chronic pulmonary disease | 254 (5.4) | 88 (5.7) | 166 (5.2) | 0.5365 |
| Connective tissue disease | 46 (1.0) | 11 (0.7) | 35 (1.1) | 0.2113 |
| Peptic Ulcer Disease | 172 (3.6) | 64 (4.1) | 108 (3.4) | 0.2151 |
| Diabetes without chronic complications | 661 (14.0) | 252 (16.3) | 409 (12.9) | 0.0020 |
| Diabetes with chronic complications | 85 (1.8) | 39 (2.5) | 46 (1.5) | 0.0138 |
| Renal disease | 98 (2.1) | 42 (2.7) | 56 (1.8) | 0.0384 |
| Hemiplegia or paraplegia | 9 (0.2) | 4 (0.3) | 5 (0.2) | 0.4739 |
| Leukemia | 9 (0.2) | 2 (0.1) | 7 (0.2) | 1.0000 |
| Lymphoma | 6 (0.1) | 2 (0.1) | 4 (0.1) | 0.6439 |
| Tumor without metastasis | 91 (1.9) | 36 (2.3) | 55 (1.7) | 0.1764 |
| Metastatic solid tumor | 11 (0.2) | 3 (0.2) | 8 (0.3) | 1.0000 |
| Mild liver disease | 63 (1.3) | 18 (1.2) | 45 (1.4) | 0.5030 |
| Moderate or severe liver disease | 7 (0.1) | 2 (0.1) | 5 (0.2) | 1.0000 |
| AIDS | 3 (0.1) | 0 (0.0) | 3 (0.1) | 0.5555 |

**Supplementary Table 2: Screening accuracy of the 5-2-1 screening criteria in identifying patients with advanced Parkinson’s disease according to H&Y stage (****H&Y 1–2 vs H&Y 3–5)**

|  | **H&Y 3-5** | | **Adjusted Model ^a^** | | |
| --- | --- | --- | --- | --- | --- |
| **Indicator** | **No** | **Yes** | **OR (95% CI)** | **Correct Classification (%) ^b^** | **AUC ^c^** |
| 5-2-1 screening criteria |  |  |  |  |  |
| Negative | 2563 | 605 | 1 | - | - |
| Positive | 575 | 971 | 4.74 (4.05, 5.55) | 78.62 | 0.84 |
| Individual clinical indicators |  |  |  |  |  |
| ≥2 h off-time/day |  |  |  |  |  |
| Negative | 2749 | 758 | 1 | - | - |
| Positive | 389 | 818 | 5.19 (4.39,6.13) | 78.83 | 0.84 |
| ≥1 h troublesome dyskinesia/day |  |  |  |  |  |
| Negative | 3110 | 1425 | 1 | - | - |
| Positive | 28 | 151 | 6.98 (4.40, 11.06) | 77.00 | 0.81 |
| ≥5 doses of oral levodopa/day |  |  |  |  |  |
| Negative | 2851 | 863 | 1 | - | - |
| Positive | 287 | 513 | 3.35 (2.78, 4.05) | 77.47 | 0.82 |

^a^ Regressions adjusted for age, sex, time since diagnosis, Charlson Comorbidity Index, and country. ^b^Correct classification is the percentage of patients correctly classified per 5-2-1 criteria (sum of true positive and true negatives divided by total number of patients). ^C^ AUC is a screening accuracy measure that balances sensitivity and specificity. AUC is interpreted as follows: AUC = 0.5, non-informative; AUC = 0.5 to ≤0.7, less accurate; AUC = 0.7 to ≤0.9, moderately accurate, AUC 0.9 to <1, highly accurate; AUC = 1, perfect test

AUC, area under the curve; CI, confidence interval; H&Y, Hoehn and Yahr stage; OR, odds ratio.

**Supplementary Table 3: Outcomes in patients with discordant classifications according to clinician judgment and 5-2-1 screening criteria**

|  | **Overall**  **(N=114)** | **Early or intermediate PD/ 5-2-1-positive**  **(N=994)** | **Advanced PD/ 5-2-1-negative**  **(N=150)** | **p-value** |
| --- | --- | --- | --- | --- |
| Patient age, years, mean (SD) | 70.5 (10.3) | 69.4 (10.2) | 77.8 (7.7) | <0.0001 |
| Age at diagnosis, years, mean (SD) | 64.1 (10.6)^a^ | 63.1 (10.5)^b^ | 71.1 (8.8)^c^ | <0.0001 |
| Number of uncontrolled motor symptoms, mean (SD) | 5.1 (3.0) | 5.1 (3.0) | 5.1 (3.1) | 0.8002 |
| Key uncontrolled motor symptoms, n (%)^d^ |  |  |  |  |
| Shuffling walk  Freezing of gait  Falling/imbalance  Lack of arm swing  Tremor at rest  Rigidity  Bradykinesia | 569 (49.7)  473 (41.3)  481 (42.0)  494 (43.2)  704 (61.5)  686 (60.0)  685 (59.9) | 490 (49.3)  409 (41.1)  399 (40.1)  445 (44.8)  623 (62.7)  592 (59.6)  599 (60.3) | 79 (52.7)  64 (42.7)  82 (54.7)  49 (32.7)  81 (54.0)  94 (62.7)  86 (57.3) | 0.4836  0.7230  0.0010  0.0060  0.0475  0.5316  0.5318 |
| Number of uncontrolled non-motor symptoms, mean (SD) | 3.2 (3.4) | 3.1 (3.2) | 3.6 (4.3) | 0.1324 |
| Key uncontrolled non-motor symptoms, n (%)^d^  Confusion  Depression/Mood  Anxiety  Poor concentration  Short-term memory loss  Difficulty planning activities  Hallucinations  Other cognitive impairment  Behavioral problems | 125 (10.9)  294 (25.7)  248 (21.7)  274 (24.0)  234 (20.5)  218 (19.1)  63 (5.5)  57 (5.0)  35 (3.1) | 94 (9.5)  258 (26.0)  222 (22.3)  234 (23.5)  185 (18.6)  175 (17.6)  46 (4.6)  38 (3.8)  22 (2.2) | 31 (20.7)  36 (24.0)  26 (17.3)  40 (26.7)  49 (32.7)  43 (28.7)  17 (11.3)  19 (12.7)  13 (8.7) | 0.0002  0.6886  0.2017  0.4122  0.0002  0.0024  0.0031  <0.0001  0.0002 |

^a^N=698; ^b^N=618; ^c^N=80; ^d^Key symptoms are presented for brevity; other motor and non-motor symptoms were selected

PD, Parkinson’s disease; SD, standard deviation.

**Supplementary Figure 1. Measures of clinical burden in H&Y stage 1–2 patients compared with H&Y stage 3–5**

| 21.2* |  | 3.3* |
| --- | --- | --- |
| 2.3* |  | 46.1* |

*p<0.0001.

ADL, activities of daily living; H&Y, Hoehn and Yahr; MMSE, Mini-Mental State Examination; PD, Parkinson’s disease; UPDRS, Unified Parkinson’s Disease Rating Scale.

**Supplementary Figure 2. Measures of healthcare resource utilization in H&Y stage 1–2 patients compared with H&Y stage 3–5**

| 18.5* |  | 3.6* |
| --- | --- | --- |
| 11.1* |  | 19.6* |

*p<0.0001.

H&Y, Hoehn and Yahr

**Supplementary Figure 3. Measures of humanistic burden in H&Y stage 1–2 patients compared with H&Y stage 3–5**

| 13.9* |  | 0.2* |
| --- | --- | --- |
| 11.9* |  | 9.6* |

*p<0.0001.

EQ-5D, EuroQol 5-Dimension; H&Y, Hoehn and Yahr; PD, Parkinson’s disease; PDQ-39, Parkinson’s Disease Questionnaire-39; ZBI, Zarit Burden Interview.
